# Assessing the industrial readiness for adoption of industry 4.0 in Nepal: A structural equation model analysis^☆^

**DOI:** 10.1016/j.heliyon.2022.e08919

**Published:** 2022-02-21

**Authors:** Sharad Rajbhandari, Niranjan Devkota, Ghanashyam Khanal, Surendra Mahato, Udaya Raj Paudel

**Affiliations:** a Quest International College, Pokhara University, Gwarko, Lalitpur, Nepal; b Morgan International College, Tribhuvan University, Kathmandu, Nepal; c Nepal Commerce Campus, Tribhuvan University, Kathmandu, Nepal

**Keywords:** Industry 4.0, Nepalese industries, Industries' readiness, Structural equation modeling

## Abstract

Industry 4.0 (I4.0) mainly focuses on manufacturing technology and processes that comprise a cyber-physical system (CPS), Internet of things (IoT), Industrial Internet of things (IIOT), Cognitive Computing and Artificial intelligence as innovation towards the computerization and exchange of information. Though it has been an emerging issue in the industrial and business world, very few studies has been undertaken in the Nepalese case. In this context, the study accesses the industrial readiness for the adoption of I4.0 in Kathmandu valley, Nepal. Explanatory Exploratory research design was adopted to examine if variables chosen actually affect industrial readiness and data was collected through structured questionnaires using both descriptive and inferential statistics via Structural Equation Modeling (SEM). Adopting the census method to collect data, a total of 287 industries located in three industrial estates were taken as a population. Collected data were analyzed using SPSS and AMOS. The study found that the major problem while adopting I4.0 is the lack of skilled manpower in the industrial sector. In addition, we found people, customers, and culture, strategy and leadership, governance and operations have a significant effect on technology innovation decision making. Similarly, government intervention plays a significant mediation role between dependent and independent variables. Furthermore, this study found industries in Nepal are not ready for I4.0 as they are not implementing any enabling technology that enables industry 4.0. These results will support managers/policymakers in recognizing the strategic actions that can be embraced in order to improve the company's readiness level to seek the optimum benefits from the adoption of I4.0 paradigms.

## 1 Introduction

The tendency directing to an increasingly the digitally transformed manufacturing environment is an emerging issue for scholars and practitioners of different arenas (Hannola et al., 2018; Richter et al., 2018). The factors affecting the attention on the impact evaluation of Industry 4.0 (I4.0) technologies for sustainable production (Bai et al., 2020; Shahid Iqbal et al., 2018) and operations management (P&OM) is explained on the basis of developing more efficient and effective value chains and simultaneously decreasing resource consumption (Marre et al., 2015).

"Industry 4.0″ has been exceedingly popular in recent times in industrial and business arenas. This term for the first time was coined in Germany (Hermann et al., 2016; Bartodziej, 2017) to define a well-proven and established initiative driving the fourth industrial revolution (Chung and Kim, 2016; Min et al., 2019) and used by experts to describe it (Siemens, 2017; Albers et al., 2016; Lu, 2017), has three distinct features that differentiate it from Industry 3.0 (Schwab, 2016): (i) the exponential pace at which technology is evolving; (ii) the breadth and depth of technological advancement, which combines multiple technologies; and (iii) the extent of the impact across the entire system, thus affecting companies, industries, and whole countries. This means that the disruptive effects of (I4.0) are widely felt at all levels, and require action to deal with their impact (Sanders et al., 2016).

The very first industrialization occurred which was triggered by the development of the steam engine in England in the 18th century. In the second half of the 19th century, the Second Revolution occurred in Europe and the USA. This transition is demonstrated by the mass processing and replacement of steam by chemical and electrical technologies (Bonilla, et at., 2018). To meet the growing demand, many innovations were introduced in industry and mechanization, such as the automated assembly line which enabled productivity to increase (Tri et al., 2021). The integrated circuit (the microchip) was the invention that contributed to the Third Industrial Revolution. To achieve greater production performance, electronics and IT are required that have taken place in many industrialized countries across the world throughout the last years of the twentieth century (Acemoglu, 2002).

Only if the government has clearly defined information and coordination advantages and can serve as a guaranteed body, the industrial policy will have positive effects and the market can relocate resources in a timely and effective fashion. In the mid-1980s, Nepal adopted transparent policies to improve international trade, eliminate possible dependency and build employment but did not meet the above objectives (Chopra et al., 2019).

Nevertheless, reliance on external markets to meet the increasing demand for capital equipment and intermediary materials hindered industrial development, given the industry's highly profitable life. Nepal has been introducing routine economic plans since the late 1950s. The poor level of household mobilization, poor outside assistance and improper project, while government expenditure has risen, has been the main cause of low production potential (Mainali, 2017). The general conviction is that the economies of liberalization improve domestic and foreign competitiveness, boosting private industry efficiency. Consequently, in the mid-1980s liberalization started with the beginning of the financial market restructuring, but in the 1990s widespread liberalization started (Mainali, 2017). CBS (2014), Nepal's Sustainable Manufacturing Efficiency Index, ranks 119th out of 135 countries (CIP). The Economic Survey (2018) found that there were 7,832 sectors. Two-thirds of the industries in Province 3 and the smallest in Karnali-Pradesh before mid-March of the financial year, 2018/19 are registered among these factories. This data indicates the sluggish speed of economic growth in Nepal and the poor industrial condition in Nepal. Liberalization began in 1985 with the introduction of financial sector legislation, and major liberalization in 1991 with the formulation of various acts relating to trade and industry.

Though, globally adopted technologies are still new as well as applied technologies are still behind than in the global context. Nepal lies between giant nations China and India which indicates a greater possibility of the economic scale. To gain such economies of scale there is the significant role of industrialization having the latest technology. Thus, research and development in the industrial sector of Nepal are essential to cope with the pace of industrial development and gain economies of scale. Despite various liberalization steps have been introduced-including elimination of import licenses, full Nepalese rupees convertibility, the opening of joint venture financing, FDI arrangement and one window scheme, new foreign Investment, and Technology Transfer Act, etc. This causes an increase in dependency on Nepal. Until now, large-scale trade has been owned by foreigners, primarily Indians. Dependency on other nations is like a huddle for Nepal's overall development.

In the age of globalization, the adoption of I4.0 is, therefore, one of the drivers that enable the industries to achieve the desired level of production minimizing the cost of production (Dachs et al., 2019). In the context of Nepal, there were several studies of study which were related to the industrial sector. But there was no research related to industry 4.0 through industry 4.0 is one of the burning issues of the global market. Therefore, we have the opportunity to explore about industrial revolution that different stakeholders could be benefited. Therefore, the study mainly tries to measure the readiness of the industrial sector for the adoption of the fourth-generation industrial revolution in Kathmandu valley. In addition, it identifies the status of industries in Kathmandu valley with the help of an industrial readiness index. We endeavor to analyze factors affecting industrial readiness for I4.0 and identify obstacles of implementing I4.0 using Readiness Index and Structural Equation Modelling, which was hoped to provide a deeper understanding of industrial readiness for adoption of I4.0.

The current study is organized as follows; section 2 presents the literature review including conceptual framework and hypotheses formulation. Section 3 discusses the research methodology. Section 4 presents results and discussion. The final section ends with conclusions and implications followed by limitations and future research.

## 2 Literature review

Industry 4.0 is all about concentrating primarily on production technologies and processes consisting of cyber-physical systems (CPS), Internet of Things (IoT) and industrial internet of stuff (IIOT) as innovation for computer and knowledge sharing (Morrar et al., 2017). Several theories like the theory of technological paradigm, theory of disruptive innovation, diffusion of innovation, porter's value chain and technology framework environment (TOE) clarifies the behavior for technology adaptation is influenced by several dimensions like organizational, cultural, and so on (Müller et al., 2018). Similarly, different models, Econometric measures and model, Julian, Daniel& Kai-Ingo Model, I4.0 transformation model, Technology acceptance model (TAM), and I4.0 maturity model framework states how several independent factors have their relationship with industry 4.0 (Davis et al., 1989).

In a survey conducted on 130 Chinese firm representatives by McKinsey's, Chinese manufacturing firms are found to have great enthusiasm and expectation for Industry 4.0, however, only 57% of them are fully prepared for I4.0 technologies. This study revealed that these Chinese data are far lower in comparison to the United States (71%) and Germany (68%). A lack of proper understanding of the value of these technologies is the main reason in many manufacturing industries. I4.0 technologies, in a real sense, are complex and integrated architecture manufacturing-information technology integration (Frank et al., 2019). It is difficult to evaluate the impact of these technologies based on standard evaluation; but additional evaluation for sustainability benefits and readiness can upsurge their strategic acceptance, though it makes the process even more multifaceted (Lucato et al., 2019). Therefore, it is still an important and open subject of research in I4.0 evaluation and industries readiness (Dalenogare et al., 2018; Raut et al., 2020).

Improved productivity is integral to each and every industrialization. I4.0 is a fresh, popularly discussed and researched technical framework which may ultimately be a fourth industrial revolution because it brings great improvements to the sectors that are appropriate to smart and potential industry sectors (Schuh et al., 2013; Durak and Saritepeci, 2018). The new trend brings combine cyber and mobile experiences via cyber-physical system CPS technology and opens up numerous possible opportunities which require companies to implement the new manufacturing framework to optimize efficiency and productivity (Zhou et al., 2015). I4.0 has tremendous potential, and through the structural change in terms of organization and market modals and manufacturing technologies, it will generate a variety of economic and social opportunities (Kagermann et al., 2013; Brixner et al., 2020).

The review of the literature makes it clear that the development of the industrial sector is important to the multidimensional aspect such as job growth, income creation, import substitution, foreign currency availability, etc (Saberi et al., 2019). Different industrial policies have been enacted for the promotion and development of the industries in Nepal, however, they have stagnated.

With regard to the 4^th^ Industrial Revolution, we enable the importance of an industry of global and domestic innovations which promote the fundamental principles of I4.0 such as the vertical and horizontal integration of production processes and industries and the inclusion of advanced technologies across the whole value chains (Schumacher et al., 2016). Table 1 below illustrates the most appropriate literature that concern or comprise the factors that affect the technology innovation decision-making of an organization.

**Table 1:** List of important literature review.

Category	Literature	Main Contribution
People, Customers and Culture	(Schumacher et al., 2016). (Kagermann et al., 2013)	• Use of consumer information • Sales/services digitalization • Customer's digital media competencies • Value of ICT in company
Strategy and Leadership	(Schumacher et al., 2016). (Kagermann et al., 2013)	• Implementation of I4.0 roadmap • Available resources for realization • Adoption of business model
Governance and operations	(Schumacher et al., 2016). (Kagermann et al., 2013)	• Digitalization of processes • Modeling and Simulation • Interdepartmental collaboration • Labor regulations for I4.0
Government Intervention	Kousar et al. (2017)	• Financial support • Encouragements • Training and workshops • Environmental regulation

***Industry 4.0 in Nepal***

The quest for industrialization in Nepal goes back to the mid-1930s when the then Rana regime formed a host of public undertakings with the State intervention (PEs). While half of the PEs were linked to development, the rest were retail, industrial, public and financial sectors. Biratnagar Jute Mills was founded in 1936, the oldest industry (Jha, 2016). It also marked the start of industrialization in Nepal. In Kathmandu, Balaju, the first industrial estate was established with the aid of the U.S. in 1960 (Industrial District Managem, 2018). Assistance in the organization of the creation of Industrial State to build facilities such as construction of drainage/section grounds, electricity, water supply borders on walls, industrial property halls, factories or other required services such as canteens, banks, clinics and post offices.

In 1990 a new hope for industrial revolution was put in motion after the revolution of multiparty democracy that was meant to create a pro-business environment conducive to the industrial boom. The early years displayed certain signs of progress, but since then the neo-liberalization policy has been entirely out of place. Blind outsourcing led to the shutdown of several profit-generating firms after they were sold to private companies at a cash price (Bohara et al., 2018). For the developing manufacturing market, the subsequent Maoist rebellion proved too costly. Rebels also destroyed critical facilities, such as telecoms, hydroelectricity, bridges and public buildings (Bohara et al., 2018). With the favorable political climate, the current federal administration has called for solid economic development. The administration has made its best efforts to lure international investors in many of the virgin economic sectors through regulations and benefits. In order to do this, Nepal's Board of Investment was established as a working entity and chaired by the Prime Minister himself. The notion of "One Province One Big Industrial Estate" is circulating in order to expand all the nations. The Government of Nepal, under the guidance of numerous donors such as the United States, India, the Netherlands and Germany, established the Eleven Industrial Estate in separate areas of the country (Industrial District Management, 2018).

The elected leaders may be good at debating technological change, but experience shows that we have completely lost out on the possibilities that the first three industrial revolutions bring. At the time of the first and second industrial revolutions, Nepal stayed relatively closed and isolated from the outside world. While electricity was introduced back in 1911 and the people of Kathmandu were fortunate enough to have seen motor cars even before roads were paved, these technologies were imported for the benefit and pleasure of an elite class (Bhattarai and Conway, 2021). The effect of economic transformations from subsequent industrial revolutions has kept the general population marginalized or left unaffected. Up to now, we remain busy juggling never stopping global transformations, constantly unfolding. Currently, the Nepalese people own more mobile phones than toilets; however, given the country's political situation, there is a greater chance that we might miss the Fourth Industrial Revolution (Manandhar, 2017).

### 2.1 Conceptual framework

Several theories are discussed which concern the influencing factors of industrial behavior for acceptance and implementation of new technology. The major theories that are considered during the study on industry 4.0 are theory of technological paradigm (Dosi, 1982), theory of disruptive innovation (Christensen et al., 2018), theory of diffusion of innovation (Rogers, 2004), Porter's value chain theory (Porter, 1985) and Technological-organizational-environment (TOE) framework (Tornatzky et al., 1990). Theory of technological paradigm (Dosi, 1982) said that technological paradigm is all about innovation system. Similarly, the theory of disruptive innovation (Christensen et al., 2018) focuses on creating a new market and new value network, so that finally disrupts an existing market and value network. Diffusion of innovation developed by Rogers (2004) talks about the individual thoughts which could influence the action as part of a social network. Moreover, Porter's value chain theory (Porter, 1985) suggests creating customer value considering that different elements of creating value are affected by the industrial revolution. Therefore, we should not forget the company's activities that promote value creation and how they can benefit from Industry 4.0. Similarly, the TOE framework Tornatzky et al. (1990) explains how a business adopts and introduces technological innovations depending on technology environments, Organizational environments and the technological environment. All these theories talk about the process and influencing factors for the implementation or adaptation of new technologies in industries.

Among these theories, the technological-organizational-environment (TOE) framework is more justified for studying Industry 4.0. TOE framework discusses the factors that influence the acceptance of technology and its probability (Abed, 2020). TOE explains the process through which technological innovation is adopted and implemented by the company and is influenced by the technological context, the organization, the environmental context (Oliveira and Martins, 2010). The technological dimension focuses on innovations that affect the implementation of the product, the organization dimension leads to the recognition of organizational characteristics and the environmental component represents the contextual factors (Henderson et al., 2012). The TOE method is used by the researchers to explore the use of various technologies, such as EDI, business 2.0, mobile reservation systems, ERP systems, e-SCM, e-commerce and ICT, etc (Lin et al., 2017). Thus, the TOE framework states the affecting factor for the acceptance of new technologies at the organizational level. It also explains how a business adopts and introduces technological innovations depending on technology environments, Organizational environment and the technological environment (Abed, 2020).

**Figure 1:**
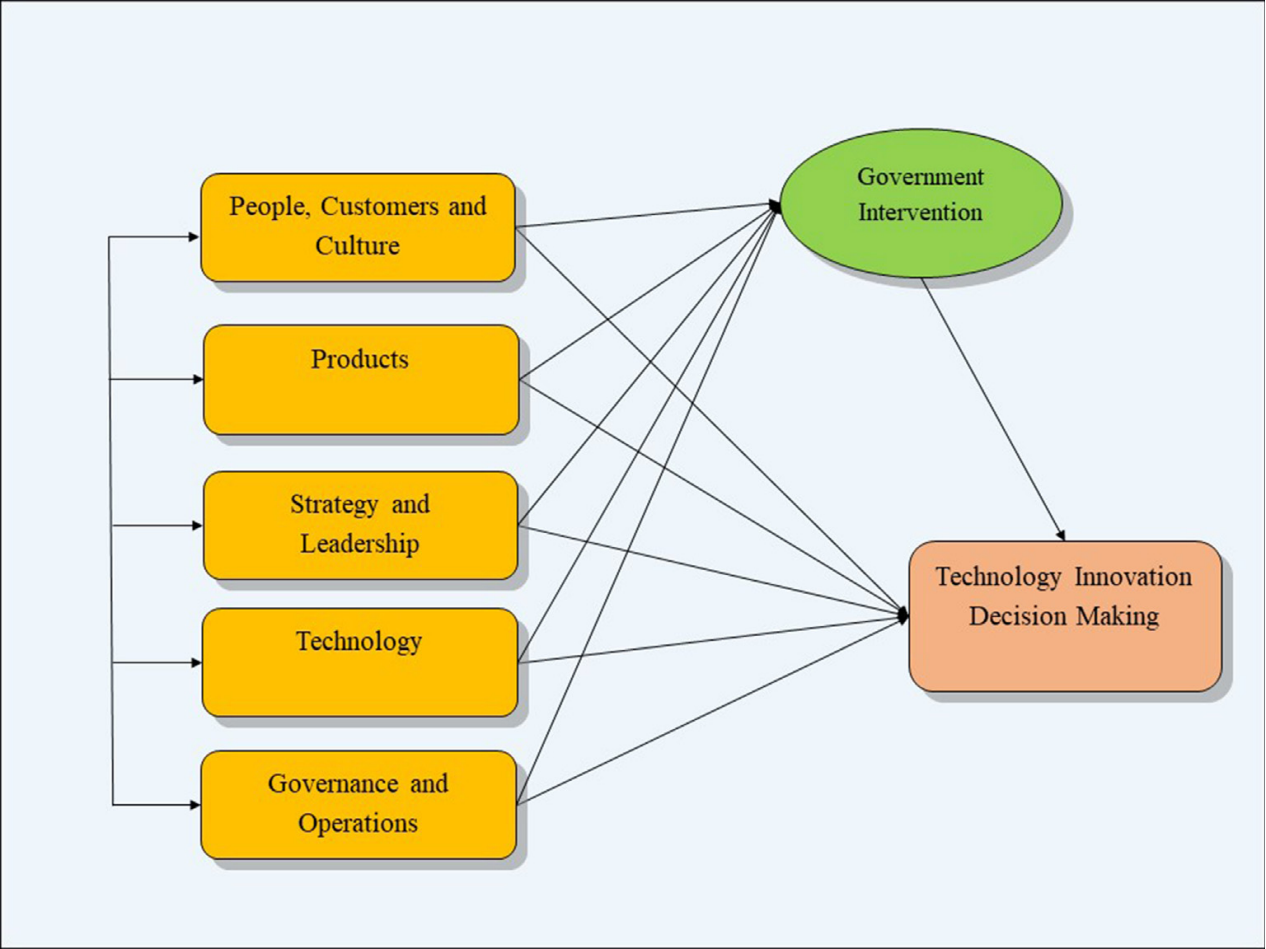
Conceptual framework. Source: Modified from Schumacher et al. (2016), Eilu (2018), Ullah et al. (2017).

In a study, Schumacher et al. (2016) developed the framework for the maturity model which is divided into 9 dimensions and 62 maturity items. For these maturity items and different dimension formulation, German government's recommendation for implementing I4.0 as well as scientific study, reports and works were used. Thus, here we modified his model and develop included five dimensions: people, custom and culture, products, strategy and leadership, technology, government and operations. Similarly, we applied the concept of Technology Organization Environment (TOE) the framework which talked about the environment that affects the technology innovation decision. And in Kousar et al. (2017), government actions are a mediation factor between environmental considerations and decision-making on technological advancement (see Figure 1).

### 2.2 Hypotheses formulation

**People Customers and Culture, and Technology Innovation Decision Making:** Technology Acceptance Model (TAM) was introduced by Davis (1986). In this study, researcher tried to theorize the implementation behavior in computer technology. This model is one of a philosophy of information systems that explains how users embrace and use technology. We want everybody to be able to use technology in the actual application of the system. We need to create Behavioral Intention, which is a factor in people, customers and culture using the technology (Davis et al., 1989).

***H***_***01***_***:****Technology Innovation Decision Making is not significantly related to People, Customers and Culture*.

**Product and Technology Innovation Decision Making:**Oesterreich and Teuteberg (2017) has mentioned that the adverse effects of wasteful and industrial use and output practices are impossible to manage in a competitive business setting. It calls for significant asset performance improvement across the worldwide economy. Several companies have therefore found opportunities to recycle goods or services and have the quality of the material and power supplies recovered over a longer time period. Thus, smart products need to be produced with the help of innovative industrial technologies.

***H***_***02***_ ***:*** *Technology Innovation Decision Making is not significantly related to Products.*

**Strategy and Leadership and Technology Innovation Decision Making:** From a strategic perspective, literature agrees that Industry 4.0 has far-reaching implications for business models (Arnold et al., 2016). These comprise both changes in established business models as well as emerging new business models. In general, the literature agrees that business model innovation is a major source of unique selling propositions and strategic differentiation, particularly in highly competitive market environments (Muller et al., 2018).

***H***_***03***_ ***:*** *Technology Innovation Decision Making is not significantly related with Strategy and Leadership.*

**Technology and Technology Innovation Decision Making:** (Machado et al. (2019)) paper leads to developments on research into industry 4.0, which recognizes that the idea of sustainable manufacturing and use of new technologies will make it possible for industry 4.0 to have a positive impact on all the dimensions of sustainability in an interconnected way.

***H***_***04***_ ***:*** *Technology Innovation Decision Making is not significantly related with Technology.*

**Governance and Operation and Technology Innovation Decision Making:** From an operational perspective, I4.0 facilitates process optimization even before value creation is realized in practice. This is mainly due to virtual simulations of production activities or even entire supply chains (Muller et al., 2018). Based on them, vertical and horizontal connection enables shorter lead times and accelerated time-to-market (Stock and Seliger, 2016). Literature has revealed that operational opportunities of new I4.0- related manufacturing technologies and production processes have a positive effect on their implementation (Oettmeier and Hofmann, 2017).

***H***_***05***_ ***:*** *Technology Innovation Decision Making is not significantly related with Governance and Operations.*

**Government Intervention and Technology Innovation Decision Making:** According to Nepal Labor Force Survey (2018–19), the unemployment rate in Nepal was estimated to be 11.4%. With this in mind, our government needs to take constructive steps and engage in making information technology education more available, along with a growing obsession with automation. Initiatives where people/organizations receive a digital workshop and are trained prepared to adapt to the changing face of technology or automation. This also enhances everybody's technological and automation knowledge (Ghimire, 2020).

***H***_***06***_ ***:*** *Government interventions mediate the relationship between Technology Innovation Decision Making and People, Customers and Culture.*

***H***_***07***_ ***:*** *Government interventions mediate the relationship between Technology Innovation Decision Making and Products. 9*

***H***_***08***_ ***:*** *Government interventions mediate the relationship between Technology Innovation Decision Making and strategy and leadership.*

***H***_***09***_ ***:*** *Government interventions mediate the relationship between Technology Innovation Decision Making and Technology.*

***H***_***10***_ ***:*** *Government interventions mediate the relationship between Technology Innovation Decision Making and governance and operations.*

## 3 Research methods

### 3.1 Research design

The study is performed by means of explanatory design as the purpose of explanatory research is to expand, extend and test theory to explain why events occur (Shmueli, 2010; Rahi, 2017). As the purpose of our study is to investigate the degree to which changes in one variable can lead to changes in the other parameters. Kivunja and Kuyini (2017) suggest explanatory research as it concentrates on explaining the relation between cause and effect and helps to answer why and how. This study aims to examine if a variable is chosen actually affects industrial readiness in the context of Industry 4.0, which is a relatively new field of inquiry in the context of Nepal.

### 3.2 Study area, population and census

The area of this study was the Kathmandu Valley of Bagmati Province, Nepal. Kathmandu valley is situated between 27°32′13″ and 27°49′10″ latitudes north and 85°11′31 ″ and 85°31′38″ longitudes to the east and 1300 m above sea level. The valley covers the entire Bhaktapur district, 50% of Lalitpur and 85% of Kathmandu. There are three industrial estates in Kathmandu Valley alone —the industrial estate of Balaju; the industrial estate of Patan and the industrial estate of Bhaktapur. In order to make use of their true potential, these industrial areas require massive infrastructure rework. The use of new technologies in the production process was one of the essential subjects. Therefore, these three industrial estates have been chosen by the researcher as a study area.

The total area occupied by Bhaktapur industrial estate was 71.28 companies and all land was fully developed, where 35 industries were in operation out of 36. The total area of Patan industrial state was 14.91 hector, 293 of which were well-formed ropanies. Similarly, there were 3.71 hector covered by the field of operations. There were currently 6 hector industries within the district, 118 of which were operating. And Balaju Industrial estate has 34 hector out of which 30 hector were well developed. There were currently 141 industries in the area, 134 of which were operating industries (Industrial District Managem, 2018). These three industrial estates include 295 enterprises, 287 of which operating. Among the industries 194 (67.6%) are small, 80 (27.9%) are medium and 13 (4.5%) are large. Also, most industries are service industries followed by manufacturing, agricultural and forest-based and construction-related industries. For our study purpose, a number of population was 287. Thus, we adopted the census method to collect data for our study.

### 3.3 Instrument and procedure for data collection

For the identification of readiness of the industries, we used a survey questionnaire. With a 5-point Likert scale, all constructs were scaled from 1 (strongly disagree) to 5 (strongly agree). A summary of the steps for the five constructs used as different variables and the framework used as a dependent variable for the application of Industry 4.0 can is shown in Table 3 below. In order to measure I4.0 total seven constructs were prepared i.e. People, customers and culture (7 items), Product (5 items), strategy and leadership (6 items), Technology (5 items), Governance and Operations (6 items), Government interventions (6 items) and Technology innovation decision making (5 items).

Industries were asked to respond to a structured questionnaire. Kobo toolbox was used to hold and fill the questionnaire. Data were collected between March–December 2020. We did census as the data collection technique and collected 287 responses from the industries and there was no case of data missing. More concretely, the collected data comprises 216 manufacturing industries, 29 service industries, 6 construction industries and 36 agriculture and forest-based industries. All the validity concerns i.e. construct, convergent and discriminant validity are taken care of, as per its suitable stage, and made to ensure that the data use has followed the issues needed for validity and reliability. Software including MS Excel, SPSS (version 21) and SPSS AMOS (version 22) is used to get results of the inferential analysis.

### 3.4 Structural Equation Modelling

In order to evaluate the hypothesis formulated in section 2, Structured Equation Modeling (SEM) was used. SEM enables the researcher to model links between many predictors and determinant factors, to construct unobserved LV's, to build model errors in measuring the observed variables, to a categorical monitor substantial/theoretical and to assess hypothesis based on quantitative understanding. SEM, therefore, includes widespread statements and developments of first-generation procedures (Chin, 1998).

This study has the purpose to access the factors that affect the technology innovation decision-making. A SEM approach is appropriate for this aim, as it provides a higher degree of sophistication needed for a deeper interpretation of the circumstances and assumptions for acceptable implementation (Chin, 1998). The key reason why SEM has become such a popular choice for analytics is that it has much strength. The ability to assess latent models offers a separate estimation of connections between latent buildings and their manifest indicators is a very well-recognized function. The accessible global fit tests are other commonly accepted assets, and a quick assessment of very complicated models involving a large number of linear equations can be given (Tomarken and Waller, 2005).

The structural equation model generally consists of measurement theory and structural equation model. The measurement models are specified as (Muthen and Asparouhov, 2012):

(1) y = Λy η + ε

(2) x = Λx ξ + δ

And, the structural equation model is specified as:

η = α + βη + Γξ +ζ

where, y = outcomes variables. x = input variables. Λy = latent variables (observed response variables). Λx = latent variables (observed response variables). ε = error. δ = error. η = latent variables (unobserved response variables). ξ = latent variables (unobserved response variables). α = vector of intercepts and β = matrix of coefficient.

## 4 Result and discussion

### 4.1 Descriptive statistics

#### 4.1.1 Socio-demographic characteristics of respondents

Among the respondents included in this survey, 78% were male and 22% were female. In a similar study conducted by B.K. et al. (2019) female participation was 24% which indicates that this study has similar female participation as compared to the previous study. The study also showed that from the total respondents (287), 216 respondents have more than 4 years of work experience, 57 respondents have 2–4 years of experience and only 14 respondents have less than 1 year of experience. This indicates that most of the respondents were experienced in their field of work. On the other hand study of B.K. et al. (2019) have shown that 3% of respondents had less than 5 years of experience and others have more than 5 years of experience.

Table 2 shows, most of the respondents have bachelor's (i.e. 153) and master's (i.e.37) degrees and works on the top level and middle level. Similarly, most of the respondents having intermediate and others education were in operating and middle level. Others in education level indicate respondents whose education level was less than intermediate and more than masters. On a similar note, most of the respondents' levels of education are bachelors as found by B.K. et al. (2019) which comply with the educational status of this study.

**Table 2:** Industry 4.0 in Nepal.

1930: Rana regime host of public undertakings with the state intervention (PEs)^1^
1936: First Company Act formed
1936: Establishment of Biratnagar Jute mill
1960: Establishment of Balaju industrial estate with the help of USA^2^
1982: Ministry of industry, commerce, and supplies formed first time as a part of Nepalese government
1988: Established Industrial District Management Limited^2^
1992: Foreign investment and technology transfer act formed
1994: Privatization Act formed
2004: Nepal become a member of the World Trade Organization
2019: Ten industrial estates are operating in different parts of Nepal^2^

Sources: 1 = Rajbhandari et al. (2020); 2 = Industrial District Managem, 2018

#### 4.1.2 Industrial readiness index for adoption of industry 4.0

This part indicates industries' preparedness for the adaptation of I4.0. Industrial readiness for the adoption of I4.0 is measured by the readiness index which includes the 15 enabling technologies for I4.0 and ranked industries readiness degree by following the readiness degree table which is developed by Pacchini et al. (2019). Enabling technologies includes Autonomous robot, Simulation, Vertical/horizontal integration, Cyber security, cloud computing, Additive manufacturing, Cyber security, Autonomous vehicles, M2M communication, Smart factory, intelligent material, Mobile computing, RFDI, Artificial intelligent and Cyber-physical system as shown in Table 3 (Pacchini et al., 2019). The study revealed that some of the industries have the digital infrastructure but 82.92% of industries don't have a digital infrastructure in their organization. Industries also revealed that only 4.88% of industries use Autonomous robots in the organizational process. Similarly, for organizational processes 3.48% of industries use Simulation. The result also states 95.47% of industries are not doing any vertical/horizontal integration. Almost all of the industries don't have any precaution for organization's data, only 4% of them have precaution for data protection. The result also shows that 98% of industries were not using Cloud computing for organization's data and 97% are not using RFID for tracking their progress in the product line.

**Table 3:** Variables and their definitions.

Construct	Items	Observed Variables	Descriptions
Peoples, Customers and Culture	PC2	Digitalization of sales/services	Organization digitalizing sales/service for buyers/customers.
	PC3	Costumer's Digital media competence	Customers are well competence with digital media.
	PC4	Value of ICT in company	Organization gives high Value towards ICT in company.
	PC5	ICT competences of employees	Employees are well competence with ICT.
Product	PR3	Product integration into other systems	Organization's Products are integrating into other systems to produce smart products.
	PR4	Differentiation of products	Organization differentiates its products on mostly by using new technology.
	PR5	Product positioning strategy	Organization product positioning strategy supports new technology adaptation.
Strategy and Leadership	SL1	Implementation I40 roadmap	Organization uses a road map for the planning of Industry 4.0 activities in enterprise.
	SL2	Available resources for realization	Organization has available resources for realization of industry 4.0.
	SL3	Adaption of business models	Organization adapts business models for industry 4.0 activities.
Technology	TE1	Existence of modern ICT	Organization has existence of advance industrial ICT to support industry 4.0.
	TE2	Utilization of mobile devices	Organization Utilize mobile devices for data transformation,
	TE4	Availability	Organization has enough technology to perform advance smart production activities.
	TE5	Characteristics	Organization's existing technology Characteristics supports industry 4.0 activities.
Governance and Operation	GO1	Decentralization of processes	Organizational activities are decentralized for new industrial technology adaptation.
	GO2	Modeling and simulation	Organization has Modeling and simulation for utilizing managerial or technical decision making.
	GO3	Interdepartmental collaboration	Organization has Interdisciplinary character to support industry 4.0
	GO4	Labor regulations for I4.0	Organization has proper Labor regulations for I40 implementation.
Government Intervention	GI1	financial support	The government provides financial support for the adaptation of new industrial technologies.
	GI2	Encouragement	The Government encourages business to produce smart product using new technologies.
	GI3	Trainings and workshops	The Government arranges Trainings and workshops to promote the use of new technologies.
Technology Innovation Decision Making	TD1	Top level supportiveness	Top level executives are supportive for new innovative technology.
	TD2	Interest of managers	Managers are interested in adopting new technology.
	TD4	Organization culture	Organization creating open culture for innovation and creativity to support technology adaptation.
	TD5	Managers' capabilities	Managers are well capable to take decision about new technology adaptation.

Notes: The items including PC1, PC6 and PC7 from construct 1; PR1 and PR2 from construct 2; SL4, SL5 and SL6 from construct 3; TE3 from construct 4; GO5 and GO6 from construct 5; GI4 and GI5 from construct 6; and TD3 from construct 7 were drop after performing Confirmatory and Explanatory Factor Analysis and these items value remains below 0.5.

Similarly, only 1.38% of industries use AI in the organization process but One-third of the industries uses Digital systems for the organization's process. Also, only a few industries use intelligent material i.e. 2.43%, Smart factory concept i.e. 2.78%, Autonomous vehicles i.e. 3.13% and M2M communication i.e. 8.36%. Thus, we can conclude that average industries have only 7.22% of knowledge about the technologies that enable the industry 4.0 in industries. This states that industries in our study area were don't know much about enabling technologies that enable industry 4.0. They were in the embryonic stage of readiness level. This means they have knowledge about less knowledge of a small number of technologies. This indicates that we should formulate policy to make them aware or knowledgeable about enabling technologies rather than going for implementation of new innovative technologies. The readiness levels of individual industries are shown in the table below:

Table 3 demonstrates that the majority of industries were in the initial stage of readiness followed by initial, primary, intermediate, and advanced. Also, none of the industries were ready for industry 4.0. The study of Castelo-Branco et al. (2019) accesses the readiness of manufacturing companies in the European Union. Castelo's study found that Scandinavian countries display a high degree of acceptance of both industry 4.0 and big data sophistication, even though their levels of adoption are not homogeneous. In our study industries' result demonstrate that industries are not ready for industry 4.0, because they are still not familiar with new innovative technologies and their policy is not supportive of the adaptation of technologies that enables industry 4.0.

### 4.2 Inferential statistics

#### 4.2.1 Data analysis

***Confirmatory factor analysis (CFA):*** CFA has been used to evaluate the discriminant validity and reliability of the constructs. Three fit indexes were deployed to assess the model fit: the comparative of fit index (CFI), Root mean square residual (RMR) and root mean square error of approximation (RMSEA). CFI value of 0.9 or more along with the RMR and RMSEA values of 0.1 or less suggests a good fit (Harrington, 2009; Brown, 2015).

CMIN/DF (chi-square statistics to degrees of freedom) is 2.809, which is generally acceptable and provides greater model support (Hair et al., 2014). The comparative fit index is 0.937, which is above the necessary minimum of 0.90 (Hair et al., 2014). The root means a square error of approximations was 0.069, which was just above the suggested level of 0.05 but below the upper limit of 0.08 (Hair et al., 2014). It means that the measurement model is fit (CMIN/DF = 2.351, P < 0.01, CF1 = 0.937, RMR = 0.069, and RMSEA = 0.069). The measure measurement models refer to implicit or explicit models that relate their indicators to the latent variable (Fornell, 1992). The research has followed Hair et al. (2014) to assess the measurement model, whereby convergent and discriminant validity were investigated. Thus, they have been summarized below:

***Convergent Validity*:** The degree of consistency with which several items can assess a single construct is known as convergent validity (CV) (Hair et al., 2014). Furthermore, CV is the degree to which particular indicators represent a construct (Urbach and Ahlemann, 2010). Hair et al. (2014) advocated that factor loadings, composite reliability (CR), and average variance extracted (AVE) have been used to assess the CV. The suggested values of AVE should be greater than 0.5, and the CR should be greater than 0.7 (Hair et al., 2014). Furthermore, the values of CR must be greater than the respective values of AVE too to meet the criteria of CV. Table 4 also shows that the result of the measurement model has outscored the recommended values (Hair et al., 2014).

**Table 4:** Respondents' education level.

Education level	Employee Level			Total
	Operating level	Middle level	Top-level	
Intermediate	15	10	28	53
Bachelors	3	64	86	153
Masters	0	1	36	37
Others	24	2	18	44
Total	**42**	**77**	**168**	**287**

***Discriminant Validity:*** By analyzing the relationship between potential redundant measures, discriminant validity refers to the degree to which predictors distinguish between constructs or measure various concepts (Ramayah et al., 2018). Furthermore, discriminant validity refers to how well items distinguish or quantify various concepts across constructs (Hair et al., 2014). The discriminant validity was assessed using the Fornell and Larcker (1981) technique, with the criterion for determining discriminatory validity being a comparison of the AVE with square correlations or the AVE square root with correlations. The second method, as given in Table 2, was used to compare the AVE square root to the correlation. When the square root of the AVE, as displayed on the diagonals, surpasses the values in the columns and rows on that specific construct, we should conclude that the measurements are discriminatory. The values in the diagonals are higher than the values in their respective columns and rows, according to Table 5. The metrics used in this study are unique and have sufficient discriminating validity.

**Table 5:** Readiness Level of individual Industries.

Degree of Readiness (DR)-%	Status	Characteristics	Industrial Stage of Readiness
0 < DR < 10%	Embryonic	The firm knows a limited range of activated innovations superficially (if any).	221 (77.00%)
10 < DR < 25%	Initial	Some innovations are known to the organization but not all of them can be known.	45 (15.68%)
25 < DR < 50	Primary	All developments are well known to the Organization, but not all have already been implemented.	13 (4.53%)
50 < DR < 75%	Intermediate	The business understands all innovations and it all started to be applied.	6 (2.09%)
75 < DR < 90%	Advance	The business has full understanding and a high degree of acceptance of all technology	2 (0.70%)
90 < DR < 100%	Ready	Almost all the activated innovations are completely embraced by the organization.	0

**Figure 2:**
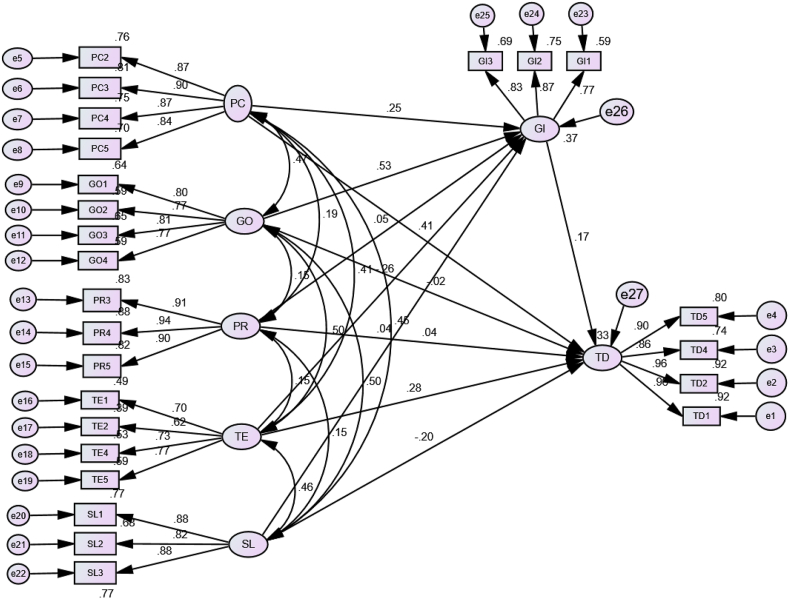
*SEM for direct, indirect and mediation relationship*.

***Structural Equation Modeling and Hypotheses testing:***Figure 2 and Table 6 show the results of testing the structural model. R2 has been used to assess the predictive strength of the structural model used for the study. R2 shows the sum of variation described by the independent variables (Barclay and Smith, 1995). Chin (1998) advocated that perhaps the R2 values of 0.67, 0.33 and 0.19 were considered significant, moderate and low or weak, respectively. Therefore, GI and TD R2 values were currently obtained to be 0.37 and 0.33, respectively, from AMOS results that provide moderate explanatory strength. For the hypothesized relationships, t-tests and path coefficients were determined using a bootstrapping approach with a sampling of 5000. Table 3 demonstrates the results of structural path model.

**Table 6:** Items loading, composite reliability and average variance explained.

Constructs	Items	Loadings	Cronbach's Alpha	Composite Reliability	Average Variance Explained
PC	PC2	0.861	0.925	0.926	0.758
	PC3	0.86			
	PC4	0.819			
	PC5	0.756			
PR	PR3	0.937	0.939	0.94	0.839
	PR4	0.945			
	PR5	0.925			
SL	SL1	0.83	0.892	0.894	0.738
	SL2	0.847			
	SL3	0.875			
TE	TE1	0.708	0.797	0.799	0.5
	TE2	0.802			
	TE4	0.719			
	TE5	0.749			
GO	GO1	0.752	0.867	0.867	0.62
	GO2	0.749			
	GO3	0.77			
	GO4	0.813			
GI	GI1	0.823	0.861	0.862	0.676
	GI2	0.805			
	GI3	0.868			
TD	TD1	0.919	0.957	0.957	0.846
	TD2	0.914			
	TD4	0.857			
	TD5	0.895			

Note (s): PC = People, Customers and Culture, SL = Strategy and Leadership, TE =, G0 = Governance and operations, GI = Government Intervention, TD = Technology innovation and Decision Making.

For the hypothesized relationships, t-tests and path coefficients were determined using a bootstrapping approach with a sampling of 5000. Table 3 demonstrates the results of structural path model. From the analysis, it was found that PC (β = 0.409∗∗∗, t-value = 5.875; P < 0.001) has significant effect on TD (H1). GO (β = 0.022, t-value = -0.247; P > 0.05) has no significant influence on TD, which does not support H2. Likewise, TD is not significantly influence by PR (β = 0.042, t-value = 0.786; P > 0.05), which does not support H3. TE has significant effect on TD (β = 0.281, t-value = 3.506; P < 0.01), which support H4. SL (β = -0.201, t-value = -2.870; P < 0.01), which supports H5. GI has positive and significant influence on TD (β = 0.172, t-value = 2.302; P < 0.05), meaning that H6 is accepted.

Table 7 reveals the mediation analysis. The bootstrapped approach was used to assess the indirect effect of independent variables on the dependent variables (TD) (Preacher and Hayes, 2004). The process had used a 95 percent bias-corrected bootstrapped confidence interval using 1000 replications to evaluate the indirect influence of IVs on TD. The coefficient associated with the indirect effect of PC via GI to TD was significantly different from zero (β = 0.044, t-value = 2.20; P < 0.05, bootstrap bias-corrected 95 percent CI = [0.014–0.118], which supports H_7_. Similarly, coefficient associated with the indirect path of TE via GI to TP was also found significantly different from zero (β = -0.084, t-value = 2.667; P < 0.05, bootstrap bias-corrected 95 per cent CI = [−0.139 to – 0.011], which supports H_10_. The result revealed that GI has significantly mediated the effect of GO on TD (β = 0.091, t-value = 3.08; P < 0.05, bootstrap bias-corrected 95 per cent CI = [0.181–0.032], which supports H_11_ (see Table 8).

**Table 7:** Results of discriminant validity (Fornel-Larcker method).

Constructs	AVE	MSV	SL	TD	PC	GO	PR	TE	GI
SL	0.738	0.249	**0.859**						
TD	0.846	0.252	0.160	**0.920**					
PC	0.758	0.252	0.452	0.502	**0.871**				
GO	0.620	0.291	0.499	0.309	0.465	**0.787**			
PR	0.839	0.038	0.150	0.156	0.194	0.149	**0.916**		
TE	0.500	0.254	0.457	0.374	0.406	0.504	0.154	**0.707**	
GI	0.676	0.291	0.302	0.314	0.415	0.539	0.140	0.135	**0.822**

**Note (s)**: PC = People, Customers and Culture, SL = Strategy and Leadership, TE =, G0 = Governance and operations, GI = Government Intervention, TD = Technology innovation and Decision Making.

**Table 8:** Results of Structural Path model of direct effects.

Hypothesized paths	Path Coefficient	S.E.	t-value	P-value	Decision
PC → TD	0.416	0.071	5.875	∗∗∗	Supported
GO → TD	-0.038	0.153	-0.247	0.805	Not Supported
PR → TD	0.048	0.062	0.786	0.432	Not supported
TE → TD	0.547	0.156	3.506	∗∗∗	Supported
SL → TD	-0.315	0.11	-2.87	0.004	Supported
PC → TD	0.254	0.11	2.302	0.021	Supported

Note(s): ∗∗∗ means significant at 0.01 level of significance, PC = People, Customers and Culture, SL = Strategy and Leadership, TE = Technology, G0 = Governance and operations, GI = Government Intervention, TD = Technology innovation and Decision Making.

Furthermore, the result has also depicted that the coefficient associated with the indirect effect of PR via GI on TD was not significantly different from zero (β = 0.009, t-value = 0.800; P > 0.05, bootstrap bias-corrected 95 per cent CI = [−0.005 – 0.029], which does not support H_8_. It means that there is no mediating effect of GI between products and technology innovation decision making. Similarly, the coefficient associated with the indirect effect of SL via GI on TD has not found significantly different from zero (β = 0.006, t-value = 0.273; P > 0.05, bootstrap bias-corrected 95 per cent CI = [−0.018 to – 0.060]. Thus, strategy and leadership (SL) do not have an indirect effect on technology innovation decision making (TD) through government intervention (GI), which does not support H_9_ (see Table 9).

**Table 9:** Results of mediation analysis.

Relationships	Path Coefficient	t-value	Confidence Interval (CI)	p-value	Decision
PC→ GI→TD	0.044	2.200	[0.014–0.118]	0.011	Supported
PR→ GI→TD	0.009	0.800	[-0.005 – 0.029]	0.336	Not supported
SL→ GI→TD	0.006	0.273	[-0.018 – 0.060]	0.574	Not supported
TE→ GI→TD	-0.084	2.667	[-0.139 to - 0.011]	0.012	Supported
GO→ GI→TD	.0910	3.080	[0.016–0.181]	0.032	Supported

## 5 Conclusions and implications

This is the first study in the Nepalese case that examines the "Industrial Readiness for adaption of Industry 4.0 ". In addition to examining the industries readiness for I4.0, we aimed to identify the status of industries in Kathmandu valley, to develop industrial readiness index for the adoption of I4.0, to analyze factors affecting industrial readiness for I4.0, to identify obstacles to implement I4.0, and to recommend a managerial solution for promoting industrial readiness for I4.0.

We found that Balaju industrial estate is the oldest industrial estate and it comprises the highest number of industries within it in comparison to other industrial estates in Kathmandu Valley. Similarly, the majority of industries in Kathmandu valley are manufacturing industries and small in scale. We created a readiness index asking 15 questions on the basis of enabling technologies of I4.0 and checked readiness level of industries by stating if 0 < DR<10% Embryonic, 10 < DR<25% Initial, 25 < DR<50% Primary, 50 < DR<75% Intermediate, 75 < DR<90% Advance and 90 < DR<100% Ready. We found that on average industries are found to have only 7% knowledge about the enabling technologies indicating they were in an embryonic stage of readiness level and have only a little knowledge about enabling technologies of I4.0.

Likewise, our results shows that industries are less aware of the activities that promote I4.0 and have a mean response of 1.32–2.58, very low dispersion. The result of this study is KMO>0.5 (i.e. 0.901 > 0.5) and BTS< 0.001 (i.e. 0.00 < 0.001, respectively) after running Bartlett and KMO. Likewise, Confirmatory Factor Analysis (CFA) ensures that data fits correctly. The study resulted in an acceptable level for all the CFA criteria. In addition, the measurement model shows the validity of the study data. All validity criteria were satisfied in this study meaning that there was no concern with validity.

In addition, people, customers, and culture, strategy and leadership, governance and operations are found to have a significant effect on technology innovation decision making. Mediation analysis was also performed to verify the mediating relationship between variables and our results showed that Government Intervention plays a significant mediation factor between dependent and independent variables. At the last of the inferential test results, hypothesis results showed that the relationships between dependent and independent variables are significant.

### 5.1 Implications for practice

After reviewing the goals, the findings and information integrated by this study are evaluated in response to feedback for stakeholders, organizations and decision-makers. First, the industries should improve the strategy implementation mechanism. 22% of industries of our study stated that the major problem was poor implementation of formulated policy and regulation. To make a more flexible environment for the adoption of new technologies, concerned stakeholders should focus on improving the strategy implementation mechanism. Improvement in custom facilities is what the government has to facilitate to industries. The major hurdles while importing technologies mentioned by industries is custom huddles. For the ease of technology implementation, the government should improve the facility of custom for industries and remove the hurdles in the best possible way.

Moreover, developing a separate mechanism for technology implementation is the need of the hour. In the context of Nepal, industries are still behind in case of the use of innovative technology. For the adoption of technologies in the industrial sector, concerned parties should develop a separate mechanism so that use of technology should ease for industries. Next, the *development of skilled manpower* is another major challenge for industries while adopting the new technologies in the organization process. Industries in Nepal lack manpower for operating new technologies. Therefore, the study will support managers/policymakers for recognizing the strategic actions that can be embraced in order to improve the company's readiness level to seek the optimum benefits from the adoption of I4.0 paradigms (Lucato et al., 2019).

### 5.2 Limitations and future research

This study focuses on identifying industrial readiness for the adoption of I4.0 in Kathmandu valley. Despite several areas covered by this study, there are some areas that need further research. This study is conducted in the industrial sector of Kathmandu Valley only, so that further research might be conducted in other industrial sector as well as other industries outside the industrial estate. Similarly, this study based on the five variables that affect the industries' decision about the implementation of new technologies. Further study might include others factors and more variables that affect the decision of industries for a more clear picture. Likewise, for the identification of readiness level, the fifteen enabling technologies were used, which might not be enough to access the readiness of industries. The future researchers might include more enabling technologies for access readiness level in clearer picture.

The future research will focus on the determination of the effectiveness of the measures incentivizing smart and sustainable manufacturing and if the Nepalese industries are ready in the adoption of the I4.0 paradigm. Likewise, future studies may be interested to map and compare the supporting measures that have been introduced by different countries around the world and compare their level of readiness and responsiveness with Nepalese industries.

## Declarations

### Author contribution statement

Sharad Raj Bhandari and Niranjan Devkota: Conceived and designed the experiments; Performed the experiments; Analyzed and interpreted the data; Contributed reagents, materials, analysis tools or data; Wrote the paper.

Surendra Mahato: Conceived and designed the experiments; Performed the experiments; Analyzed and interpreted the data; Wrote the paper.

Ghanashyam Khanal and Udaya Raj Paudel: Conceived and designed the experiments; Contributed reagents, materials, analysis tools or data; Wrote the paper.

### Funding statement

This research did not receive any specific grant from funding agencies in the public, commercial, or not-for-profit sectors.

### Data availability statement

Data will be made available on request.

### Declaration of interests statement

The authors declare no conflict of interest.

### Additional information

No additional information is available for this paper.
